# Behavioural switching during oscillations of intracellular Ca^2+^ concentration in free-swimming human sperm

**DOI:** 10.1530/RAF-21-0001

**Published:** 2021-03-09

**Authors:** Elis Torrezan-Nitao, Héctor Guidobaldi, Laura Giojalas, Christopher Barratt, Stephen Publicover

**Affiliations:** 1 School of Biosciences, University of Birmingham, Birmingham, UK; 2 Centro de Biología Celular y Molecular, Facultad de Ciencias Exactas, Físicas y Naturales (FCEFN), Universidad Nacional de Córdoba (UNC), Córdoba, Argentina; 3 Instituto de Investigaciones Biológicas y Tecnológicas, Consejo de Investigaciones Científicas y Técnicas UNC, (CONICET), Córdoba, Argentina; 4 Reproductive and Developmental Biology, School of Medicine, Ninewells Hospital and Medical School, University of Dundee, Dundee, UK; 5 Assisted Conception Unit, Ninewells Hospital Dundee, Dundee, UK; 6 Centre for Human Reproductive Science, University of Birmingham, Birmingham, UK

## Abstract

A human sperm must swim to the egg to fertilise it. To do this the sperm uses different types of swimming (behaviours) as they are needed. When we watch sperm swimming we see that they regularly change behaviour, sometimes repeatedly switching between two different types. Calcium ions inside cells are crucial in controlling many cell functions and in sperm they play a key role in regulating their behaviour. Here we have measured the concentration of calcium ions inside swimming human sperm. We found that in 12/35 (34%) of the cells we assessed, the concentration of calcium changed repeatedly, averaging more than one cycle of rise and fall per minute. These changes in the concentration of calcium ions occurred as the sperm switched swimming stroke, suggesting that oscillation of calcium concentration is involved in controlling the switching of sperm behaviour. Impaired sperm motility is an important cause of subfertility in men. Understanding how sperm behaviour is controlled will allow the development of treatments that can rescue the fertility of sperm with impaired motility.

Repetitive [Ca^2+^]_i_ transients (oscillations) have been observed by ourselves and others in unstimulated and progesterone (P4)-stimulated, immobilised human sperm (Harper *et al.* 2004, Sanchez-Cardenas *et al.* 2014). In P4-stimulated cells, these [Ca^2+^]_i_ oscillations reversibly modify flagellar beating (Harper *et al.* 2004). Since free-swimming sperm regularly and reversibly ‘switch’ their behaviour (Achikanu *et al.* 2019), we hypothesised that such oscillations also occur in P4-stimulated free-swimming sperm and regulate, or contribute to, behavioural switching.

Human sperm (consenting donors; ethical approval ERN-12-0570) were incubated under capacitating conditions (Achikanu *et al.* 2019), loaded with Fluo4 and suspended in supplemented Earle’s balanced salt solution containing 3 µM P4. Cells were viewed in an observation chamber (20 µm depth; 31°C). Fluorescence images (excitation/emission 485/520 nm, 10 Hz) were analysed as described previously (Harper *et al.* 2004, Achikanu *et al.* 2019). Recorded fluorescence is primarily from the head/neck, though oscillations initiate in the flagellum (Torrezan-Nitao *et al.* 2020).

Frame–frame variation in fluorescence intensity significantly exceeded that seen in P4-stimulated immobilised cells, consistent with the continuous rotation of free-swimming human sperm (Schiffer *et al.* 2020). Mean coefficient of variation (CV, calculated using moving, five-point samples across the duration of the recording) was 0.12 ± 0.03 (mean ± s.e.m.; *n*  = 7 cells) compared to 0.03 ± 0.02 (*n*  = 7) in immobilised cells (*P* < 0.005). Superimposed on this ‘noise’, we observed oscillations or irregular spiking of [Ca^2+^]_i_ in 34% of motile, free-swimming cells (12/35; three donors), similar to equivalent observations in immobilised cells (29.8%; 17/57; *P* = 0.65; χ^2^). Frequency of [Ca^2+^]_i_ spikes also resembled that in immobilised cells (1.03 ± 0.07/min and 1.09 ± 0.05/min ,respectively; *P* = 0.60; *t*-test).

In 18 cells, sperm tracks were generated using the position of the sperm head in each video frame. Since the video frame rate (10 Hz) was too low for calculation of standard CASA parameters, we used fractal dimension (FD) to assess the complexity of the sperm path (Achikanu *et al.* 2019). In six cells where large [Ca^2+^]_i_ spikes occurred, we saw behavioural transitions and rapid changes in FD that were associated with [Ca^2+^]_i_ signals. Figure 1A, B, C and D shows such a cell, where three periods of elevated FD occurred, each associated with an increase in [Ca^2+^]_i_ (Fig. 1D). CV of the fluorescence signal was not related to fluorescence intensity (Fig. 1C), indicating that increased fluorescence was not an artefact caused by extravagant cell behaviour during the periods of increased FD. In contrast, in six cells where paths were circular or looping, there was little variation in FD and negligible [Ca^2+^]_i_ spiking (Fig. 1E, F, G and H). In three of the other six cells we saw association of [Ca^2+^]_i_ with average path velocity, but no clear correlation with FD.

**Figure 1:**
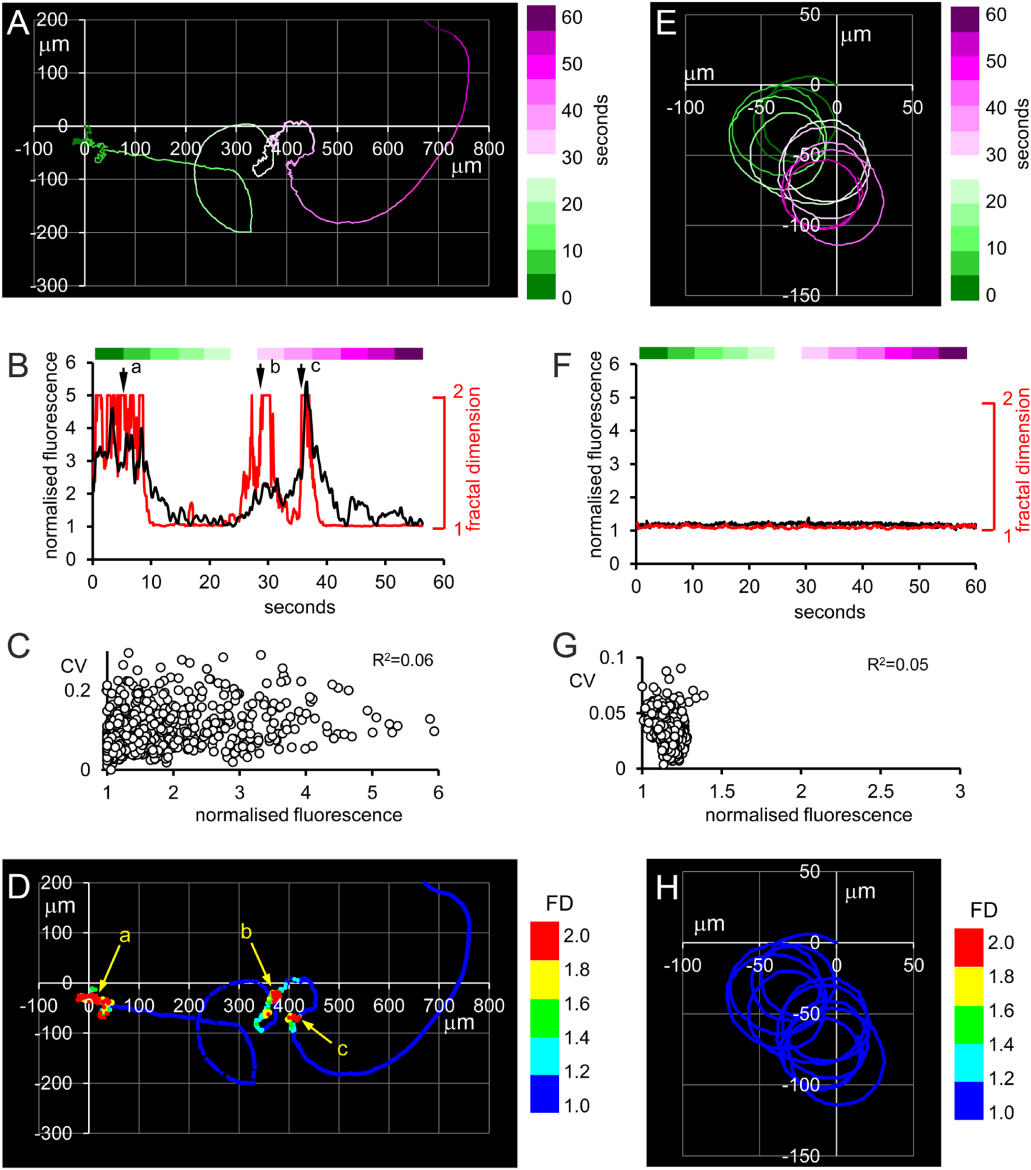
Panels A, B, C and D show data from a cell where [Ca^2+^]_i_ transients are associated with increased fractal dimension (FD). Panel A shows the 60 s sperm track with colour coding to indicate time (shown by the scale to the right of the plot). Panel B shows the time-course of [Ca^2+^]_i_ (fluo4 fluorescence; black) and FD (red). Data are normalised to their minimum value. Bar above the plot shows colour coding of time for comparison with panel A. Arrows (labelled a, b and c) indicate three periods of high FD for comparison with panel D. Panel C shows the relationship between mean fluorescence intensity and its coefficient of variation (CV), calculated over the duration of the recording by using a moving five-point sample. CV did not change when fluorescence increased. Panel D shows the sperm track colour coded to show FD (1≤FD≤1.2 (dark blue); 1.2 < FD ≤ 1.4 (light blue); 1.4 < FD ≤ 1.6 (green); 1.6 < FD ≤ 1.8 (yellow); 1.8 < FD ≤ 2.0 (red). Arrows (labelled a, b and c) indicate three periods of high FD associated with increased [Ca^2+^]_i_. Panels E, F, G and H show data from a cell with a looping path, where both [Ca^2+^]_i_ and FD remained constant throughout the 60 s recording. Details of data presentation are as for panels A, B, C and D.

We conclude that free-swimming human sperm show [Ca^2+^]_i_ oscillations/repetitive spiking similar to that observed in immobilised cells. Furthermore, these [Ca^2+^]_i_ signals are frequently associated with changes in FD. However, these are preliminary data and further investigation is required. In particular, higher frame rates (≥60 Hz; Mortimer *et al.* 1988) should be used to permit detailed analysis of the relationship between amplitude/shape of [Ca^2+^]_i_ signals and cell behaviours.

## Declaration of interest

The authors declare that there is no conflict of interest that could be perceived as prejudicing the impartiality of this article.

## Funding

Medical Research Council http://dx.doi.org/10.13039/501100000265 (MR/M012492/1) and CAPES Foundation (E T-N).

## Author contribution statement

E T-N did the experiments, E T-N, S J P and H A G analysed the data, S J P drafted the ms, all authors edited/approved the ms.
